# Temporal Influence of Transport Length on Epidermal Barrier Integrity, Mucin Transcription, and Goblet Cell Kinetics in Red Tilapia (*Oreochromis* sp.) Fry

**DOI:** 10.3390/ani16162539

**Published:** 2026-08-14

**Authors:** Hernán Antonio Alzate-Díaz, Samir Julián Calvo-Cardona, Sandra Clemencia Pardo-Carrasco

**Affiliations:** 1 Department of Animal Production, Faculty of Agricultural Sciences, Universidad Nacional de Colombia, Medellín 050034, Colombia; haalzated@unal.edu.co; 2 Veterinary Medicine and Animal Science, Faculty of Health Sciences, Universidad Tecnológica de Pereira, Pereira 660003, Colombia; samir.calvo@utp.edu.co

**Keywords:** stress, skin, goblet cells, gene expression

## Abstract

Red tilapia (*Oreochromis* sp.) fry are routinely transported between hatcheries and grow out farms, yet the effects of transport duration on epidermal barrier-associated responses and goblet cells remain poorly defined. This study assessed the impact of simulated transport on epidermal morphology, immune-related gene expression and goblet cell density in red tilapia fry. Fish were assigned to four groups (0, 5, 12 and 20 h of transport). Skin samples were analyzed for relative mRNA expression of 10 genes associated with tight junction proteins, mucins, antimicrobial peptides and cytokines using RT-qPCR, and for epidermal goblet cell density by histology. Transport significantly modulated eight genes, forming three co-expression clusters associated with inflammatory, barrier-related, and mucus-related responses, with mainly non-linear time courses. MUC2, DB-4, IL-1β, and TGF-β1b showed transient increases at 5–12 h, whereas MUC5AC and claudin transcripts increased earlier and approached control levels at 20 h. Goblet cell density remained relatively stable up to 12 h but declined after 20 h, coinciding with changes in MUC2 and MUC5AC expression and suggesting altered mucosal responses during prolonged transport. Overall, transport durations ≤ 12 h were associated with relatively stable epidermal responses, whereas 20 h induced pronounced changes in goblet cell density and barrier-associated gene expression. These findings provide information that may assist future optimization of transport practices in tilapia aquaculture. Transport stress in red tilapia fry induces rapid defensive responses, significantly modulating gene expression and mucosal dynamics; this is characterized by increased mucus production, goblet cell density, and early upregulation of mucins (MUC2), followed by later increases in tight junction genes and anti-inflammatory cytokines, alongside a temporary downregulation of antimicrobial peptides.

## Simple Summary

Red tilapia fry are often transported from hatcheries to grow-out farms, but prolonged transport can affect skin-related protective responses, which are important components of the fish’s first line of defence against environmental challenges. This study examined how different transport times affect the skin of red tilapia fry, with particular attention to genes involved in mucus production and the cells that help protect the body surface. The results showed that transport triggered early changes in genes related to skin defence and barrier-associated processes during the first 5 to 12 h. During this period, the number of mucus-producing cells in the skin remained stable. However, after 20 h of transport, these protective responses declined and the number of these cells fell markedly, indicating changes in skin-associated protective processes during prolonged transport. These findings suggest that shorter transport periods are better tolerated, whereas prolonged transport may alter mechanisms involved in maintaining skin protection. This information is valuable for fish farmers because it can help improve animal welfare, reduce losses after stocking, and support more sustainable tilapia production.

## 1. Introduction

In Colombia, Nile and red tilapia account for 56% of national fish production [1] for domestic consumption and exports to the United States, where Colombia is the second exporter [2]. Production systems range from AREL (aquaculture with scarce and limited resources) to large industrial operations such as earthen ponds, biofloc systems, reservoir cages, and in-pond raceway systems (IPRS). A large proportion of these producers, especially smaller farms, do not produce their own seed but instead purchase it from distant hatcheries, requiring land transportation.

Buyers report high post-stocking mortality rates, mainly attributed to bacterial and viral infections in fry and juveniles, especially in those already stressed by transport [3]. The fry stage represents a critical production bottleneck because immature immune competence, ongoing epidermal barrier development, and limited physiological reserves may increase susceptibility to transport-associated disturbances.

Transport in fish elicits a classical stress response that can be understood at primary, secondary, and tertiary levels. At the primary level, transport-related handling, crowding, vibration, oxygen limitation, and water-quality deterioration activate the brain–sympathetic–chromaffin and hypothalamic–pituitary–interrenal axes, leading to catecholamine and cortisol release [4,5]. At the secondary level, these endocrine responses are accompanied by metabolic and hydromineral disturbances, including hyperglycemia, ionic imbalance, oxidative stress, and altered antioxidant defenses [4,5,6]. If stress is prolonged or intense, tertiary consequences may emerge, including tissue damage, impaired immune function, reduced performance, and increased mortality risk [6,7,8].

Tacchi et al. [9] demonstrated that transport-associated stress induces changes in the epidermal mucous layer and alters gene expression, suggesting modulation of skin immune-related responses that may influence mucosal defence capacity [9]. Furthermore, Hong et al. [10] reported that high fish densities during transport not only affect water quality but also induce physiological responses that can cause skin lesions and compromise skin integrity, underscoring the need to properly manage transport conditions to support mucosal integrity in fry during transport [10]. In addition to stocking density, transport duration also affects survival, but its impact on skin-related gene expression remains poorly understood.

Evaluating genes associated with stress responses, immune regulation, and epithelial barrier function in tilapia may provide molecular indicators of stress-induced physiological changes and may help improve transport management practices. Real-time or quantitative PCR (RT-qPCR) is commonly used to quantify the expression of specific genes associated with immune response, stress, and welfare in tilapia [11].

Given the critical role of mucus as a primary defensive barrier, the effect of transport on mucus-secreting cells in the skin was also considered. Mucus functions as an essential physical and chemical barrier, protecting epithelial surfaces against pathogens and other harmful agents, and its production is primarily mediated by goblet cells [12].

Fish skin mucus is a dynamic protective layer produced by epidermal secretory cells and constitutes both a physical and biochemical barrier. Its defensive function depends on multiple components, including mucins, antimicrobial peptides, soluble immune factors, and signaling molecules that contribute to pathogen neutralization, barrier homeostasis, and modulation of inflammation [8,13,14]. Mucosal tissues are also immunological interfaces, and cytokine responses at these sites are important for activating local immune cells and recruiting leukocytes during challenge [14,15,16]. In parallel, transport or other stressors can alter mucus secretion, mucus-cell dynamics, and skin immune activity, indicating that the cutaneous mucosa is a sensitive target of transport-induced stress [8].

Furthermore, mucus regulates the selective transport of molecules and microorganisms, and its structure and composition can be modified in response to external stimuli, including transport-induced stress [7,15]. Such changes in the quantity and type of mucus-secreting cells reflect activated defense mechanisms aimed at preserving the integrity of the mucus barrier during transport [7,15].

Within this framework, the molecular markers evaluated in this study were selected to represent complementary dimensions of cutaneous mucosal defense under transport stress. MUC2 and MUC5AC were included as markers of mucus-associated secretory activity; β-defensin 4 (DB-4) as a marker associated with antimicrobial defence pathways; IL-1β, IL-6, and TGF-β isoforms as indicators of inflammatory and immunoregulatory signaling; and claudins as indicators of transcriptional regulation associated with epithelial junction processes and cell-junction function [8,13,16]. This marker set is biologically relevant because transport has been associated with altered mucin expression, antimicrobial peptide responses, cytokine regulation, and mucus-cell dynamics in tilapia and other teleosts [8,15,16]. However, despite increasing evidence that transport alters mucosal and immune responses in fish, integrated information on transport duration, epidermal goblet-cell density, and cutaneous mucosal gene-expression patterns in red tilapia fry remains limited [8,14].

Therefore, this study aimed to characterize the time-dependent effects of transport duration on epidermal gene expression profiles and goblet cell density in red tilapia fry, integrating molecular and histological approaches to evaluate cutaneous mucosal responses.

## 2. Materials and Methods

Red tilapia (*Oreochromis* sp.) fry was obtained from a commercial breeding farm in the department of Arauca, Colombia. Four treatments were compared: a control group sampled at 0 h prior to transport and three treatment groups subjected to simulated transport for 5 h, 12 h, and 20 h. Six bags were used for each treatment; subsequently, samples were randomly selected from each bag for each analysis. Each experimental unit consisted of a transport bag containing 250 fry (0.6 ± 0.2 g mean body weight). The control group provided reference values for comparing changes in gene expression profiles and histopathological parameters, specifically epidermal goblet cell density assessed by H&E staining.

### 2.1. Samples for Gene Expression Analysis

Following each transport treatment, all fish from the transport bags assigned to the same treatment were randomly mixed before sampling. Subsequently, individuals were randomly selected to generate pooled biological samples consisting of four fry per pool for gene expression analyses. This procedure was adopted to obtain sufficient RNA for RT-qPCR while minimizing potential bag-specific effects. Consequently, each biological replicate represented a random combination of individuals originating from different transport bags within the same treatment, thereby avoiding bag identity as a source of experimental variation. Under this sampling design, transport duration was considered the only experimental factor included in the statistical analyses. For histopathological analyses, a total of 350 individual fry was processed, corresponding to 70 biological samples distributed across the four transport treatments. Each biological replicate consisted of one individual fish.

All fish were euthanized by hypothermia (thermal shock at 2 °C), following institutional animal welfare and ethical guidelines. Death was confirmed before collecting skin tissue from the caudal region using an oblique incision. The number of biological replicates was established according to the minimum tissue and RNA requirements of the genetics and histopathology laboratories and following methodological approaches previously reported for studies on larval and juvenile fish [4].

Fish were transported under commercial conditions routinely used by local producers, using sealed plastic bags containing approximately 2.5 L of water and 250 fry per bag. Physicochemical water quality variables, including temperature, dissolved oxygen, pH, and salinity, were monitored at the beginning and end of each transport period using calibrated multiparameter probes and wireless data loggers. Initial and final water quality measurements for each transport treatment have been previously reported by Alzate-Díaz et al. [17] and are presented here to characterize the transport conditions rather than as experimental treatments.

### 2.2. qPCR Conditions

RNA concentration and purity were determined using a NanoDrop spectrophotometer (Thermo Fisher Scientific, Waltham, MA, USA). Only RNA samples with A260/A280 ratios ranging from 1.96 to 2.08 and A260/A230 ratios ranging from 2.01 to 2.34 were used for downstream RT-qPCR analyses. RNA integrity was further verified by electrophoresis on a 1% agarose gel by visualization of intact 28S and 18S rRNA bands. Relative gene expression was quantified by reverse transcription quantitative PCR (RT-qPCR) using the qMAXSen™ One-Step Green RT-qPCR kit (Low ROX™) (Canvax Reagents S.L.U., Valladolid, Spain). Each 20 µL reaction mixture contained 5 µL of 4× One-Step Green RT-qPCR Master Mix, 1 µL of RT Mix, RNA template, nuclease-free water, and 100 nM of each primer. Thermal cycling conditions consisted of reverse transcription at 50 °C (10 min) and initial polymerase activation at 95 °C (3 min), followed by 40 cycles of denaturation at 95 °C (10 s) and annealing/extension at 60 °C (30 s). Specificity was confirmed by melt curve analysis. Amplification was performed on a Roche real-time PCR platform. PCR efficiency for each primer set was estimated from raw fluorescence data using LinRegPCR v2021.2.; all assays exhibited efficiencies > 1.80, indicating consistent and acceptable performance. Transcript abundance was calculated using the 2^−ΔΔCt^ method with β-actin as the internal reference and the 0 h group as the calibrator. Statistical analyses were subsequently performed on log_2_-transformed relative expression values.

### 2.3. Analysis of Stress-Related Gene Expression

To preserve RNA integrity, all procedures were carried out under aseptic conditions. Tissue for RNA extraction was placed in 2 mL vials containing an RNA stabilization reagent (RNA Safeguard Reagent BSC54M1, Gentech Biosciences SAS, Bogotá, Colombia). Tissue for histopathological analysis was fixed in 3% buffered formalin prepared with sodium phosphate monobasic monohydrate (4 g/L) and sodium phosphate dibasic anhydrous (6.5 g/L).

For gene expression analysis, a housekeeping gene (β-actin) was used as the reference control in qPCR, and the expression of specific stress-related genes was quantified. Target genes included tight junction–related genes (claudin 7, claudin 8d, and claudin 12), mucin genes (MUC2 and MUC5AC), an antimicrobial peptide gene (DB-4), and cytokine genes (IL-1β, IL-6, TGF-β1a, and TGF-β1b). Gene expression was quantified by real-time PCR (qPCR) using gene-specific primers, following the protocol described by Tacchi et al. [9]. The primer sets used to quantify the mRNA levels of the selected genes were designed based on *Oreochromis* sp.; the corresponding sequences are listed in (Table 1).

β-actin was selected as the endogenous reference gene because it has been widely used in RT-qPCR studies in teleost fish [18,19,20]. However, because the stability of reference genes may vary according to tissue type and experimental conditions, its expression stability was experimentally evaluated in the present study before being used for normalization, following current recommendations for quantitative gene expression analyses [21,22]. The validation procedure and statistical results are described in Section 2.4.

Skin samples were processed and stained with hematoxylin–eosin (H&E) to evaluate key histological features, including epidermal goblet cell density.

The gene-expression dataset used for RT-qPCR analyses is available in Supplementary Table S7. The animal-level dataset data are available in Supplementary Table S6.

### 2.4. Reference Gene Validation

Before relative quantification, the expression stability of the endogenous reference gene (β-actin) was evaluated across the four transport treatments (0, 5, 12, and 20 h). Raw Ct values were first tested for normality using the Shapiro–Wilk test and subsequently analyzed by one-way analysis of variance (ANOVA) to determine whether transport duration affected β-actin expression. Because no significant differences were detected among treatments (*p* > 0.05), β-actin was considered a stable endogenous reference gene and was therefore used to normalize target gene expression using the 2^−ΔΔCt^ method.

### 2.5. Principal Component Analysis

To evaluate global transcriptional patterns across the ten target genes, a principal component analysis (PCA) was conducted using log_2_-transformed relative quantification data. Prior to multivariate modeling, all variables underwent unit variance scaling and centering (z-score normalization) to prevent bias stemming from differences in expression magnitude. The analysis was implemented in R (version 4.5.2) utilizing the FactoMineR package, with graphical outputs rendered via factoextra. To maintain the integrity of the multivariate dataset for PCA calculation, the mean value of the corresponding gene was used to impute the single missing IL-6 observation.

### 2.6. Statistical Analysis

Gene expression data were analyzed using one-way linear models, with treatment time included as a fixed effect, according to the following model:

$$Y_{ijk}=\mu +\tau_{i}+e_{ijk}$$

where *Y_ij_**_k_*** is the RQ response variable for each gene and goblet cell production, μ is the overall mean of the response variable, ***τ_j_*** is the treatment factor, where (*i* = 4) (0, 5, 12, and 20 h of transport), and $e_{\mathrm{ijk}}$ is the experimental error.

For genes satisfying the assumption of homogeneity of variances, pairwise comparisons among treatments were conducted using Tukey’s honestly significant difference (HSD) test. Pearson correlation coefficients were calculated, and only correlations remaining significant after Benjamini–Hochberg false discovery rate (FDR) correction were retained for network visualization.

To evaluate temporal changes in gene expression across the sampling times (0, 5, 12, and 20 h), orthogonal polynomial contrasts were performed using the actual sampling times as contrast coefficients. Linear, quadratic, and cubic components were evaluated for genes showing a significant treatment effect. These polynomial contrasts were used exclusively to describe statistical temporal patterns in the data and should not be interpreted as definitive biological trajectories or underlying biological processes, particularly for higher-order (cubic) trends, which are more sensitive to individual variability.

Hierarchical clustering based on correlation distances $\left(r\right)$ was used to identify co-expression modules among significantly regulated genes.

All ANOVA assumptions were validated for the residuals ${\varepsilon_{i}\;\sim \;N(0,\sigma }_{e}^{2})$; when these assumptions were not met, non-parametric analyses were used for the corresponding variables.

The full statistical workflow for gene-expression analyses is provided in Supplementary Tables S2 and S3.

### 2.7. Samples for Goblet Cell Analysis

For the histopathological analysis, each biological replicate corresponded to a single fry. Tissue samples were collected, processed, stained, and analyzed individually, ensuring that each fish represented an independent experimental unit. To obtain a representative estimate of goblet cell density within each specimen, multiple non-overlapping microscopic fields were examined from the same histological section. These fields were considered repeated technical observations rather than independent biological replicates. Accordingly, goblet cell counts obtained from the different microscopic fields were averaged for each individual fish, and all statistical analyses were performed using these fish-level values. The total number of microscopic fields examined (67, 90, 84, and 56 fields for the 0, 5, 12, and 20 h groups, respectively; 297 fields in total) is reported solely to describe the extent of the histological assessment and should not be interpreted as the experimental sample size or as independent observations. Thus, the biological replication and statistical inference were based exclusively on individual fish, while the multiple microscopic fields served only to improve the precision and representativeness of the histopathological measurements.

### 2.8. Histological Processing and Goblet Cell Quantification

Epidermal samples were collected immediately after each transport period and processed for routine histological evaluation. Histological samples were routinely processed, embedded in paraffin, sectioned at 3 μm, and stained with hematoxylin and eosin. For each fish, two histological sections, obtained from ventral and lateral skin fragments, were evaluated. Goblet cell density was quantified at 400× magnification using an Olympus BX43 series light microscope, where each microscopic field represented an area of 0.237 mm^2^. Digital images were acquired using the same microscope and analyzed with the cellSens software v4.5. (Olympus Corporation, Tokyo, Japan). All image analyses and goblet cell counts were performed under blind conditions, with the observer unaware of the corresponding transport treatment, thereby minimizing potential observer bias. The average goblet cell density calculated for each fish was used as the biological replicate for all statistical analyses. Therefore, the mean goblet cell density for each individual fish was used for all statistical analyses.

Goblet cell density was quantified as the number of goblet cells per microscopic field (cells field^−1^). Both goblet cell count and goblet cell density were initially assessed; however, because these variables exhibited near-perfect collinearity (Spearman’s ρ = 0.976, *p* < 0.001), goblet cell density was selected as the primary response variable for subsequent analyses, given its independence from section geometry and normalization to field area. A total of 297 observations were included in the study, distributed as follows: 0 h (*n* = 67), 5 h (*n* = 90), 12 h (*n* = 77), and 20 h (*n* = 63).

Prior to inferential analyses, model assumptions were evaluated. Normality within each treatment group was tested using the Shapiro–Wilk test, and homogeneity of variances was assessed using Levene’s test based on the group median. Because normality was violated in all experimental groups (Shapiro–Wilk, *p* < 0.001), a non-parametric approach was adopted. Differences in goblet cell density among transport durations were analyzed using the Kruskal–Wallis one-way analysis of variance by ranks.

Effect size was estimated using epsilon squared:

$$\varepsilon^{2}=\frac{H-k+1}{n-k}$$

where *H* denotes the Kruskal–Wallis test statistic, ***k*** is the number of groups, and ***n*** is the total sample size. Effect sizes were interpreted according to conventional thresholds: small ($\varepsilon^{2}$ ≥ 0.01), medium ($\varepsilon^{2}$ ≥ 0.06), and large ($\varepsilon^{2}$ ≥ 0.14). When significant differences were detected, pairwise comparisons among groups were conducted using Dunn’s post hoc test with Bonferroni adjustment for multiple testing. Statistical significance was set at *p* < 0.05.

The raw histopathology datasets are available in Supplementary Tables S5 and S8 and the histological workflow and goblet cell quantification report are provided in Supplementary Table S1.

### 2.9. Statistical Software

All statistical analyses were performed in R version 4.5.2 [21] using the packages tidyverse, FactoMineR, factoextra, emmeans, rstatix, car, and ggraph, ggplot2, and dunn.test.

## 3. Results

### 3.1. Water Quality

Table 2 presents the results obtained regarding water quality. As mentioned in the methodology, these results are presented in the article by Alzate-Diaz et al. [17]. Oxygen levels increased during transport due to oxygen supplementation, whereas pH declined over time, likely reflecting CO_2_ accumulation and progressive acidification in the closed transport environment. However, pH levels decreased in all treatments, with final values lower than initial ones, indicating progressive water acidification over time, highlighting environmental changes occurring during transport that may contribute to physiological stress responses. Water quality parameters measured during transport are presented in Table 2 to characterize the environmental conditions experienced by the fish during each transport duration.

### 3.2. Validation of the Endogenous Reference Gene

The stability of β-actin expression was assessed by comparing raw Ct values among the four transport treatments. Mean Ct values showed only minor variation throughout the experimental period, ranging from 17.2 ± 2.20 at 0 h to 18.6 ± 1.06 at 20 h. Raw Ct values were normally distributed within each treatment according to the Shapiro–Wilk test (all *p* > 0.05). One-way ANOVA revealed no significant effect of transport duration on β-actin expression (F(3,42) = 2.28, *p* = 0.093), indicating that β-actin expression remained unaffected by the experimental treatments. These results confirm the expression stability of β-actin under the experimental conditions and support its use as the endogenous reference gene for normalization of relative gene expression using the 2^−ΔΔCt^ method.

### 3.3. Principal Component Analysis (PCA)

The first two principal components (PC1 and PC2) explained 71.6% of the total variance in the gene expression dataset, indicating that most of the variation among samples was represented within a two-dimensional space. PC1 accounted for the largest proportion of the variance, whereas PC2 explained an additional and independent proportion of the variation in the dataset.

**Figure 1:**
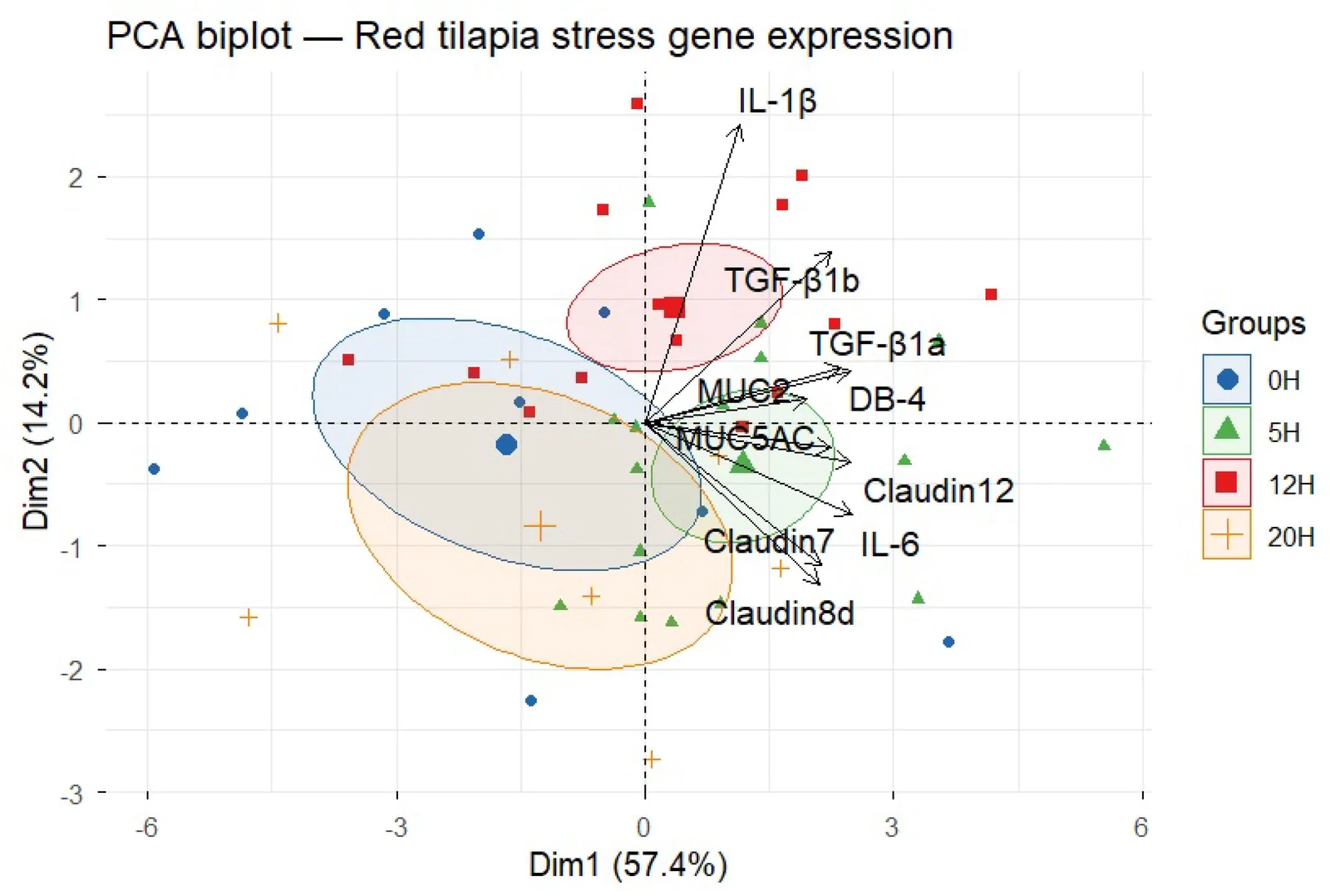
PCA biplot of individual fish coloured by treatment group with 95% confidence ellipses. PC1 primarily separates groups according to overall gene expression intensity, whereas PC2 differentiates the inflammatory axis (IL-1β, TGF-β1b) from the tight junction/mucus axis (claudins, MUC5AC).

Principal component analysis revealed differences in the multivariate gene expression profiles among transport treatments. The PCA biplot showed partial separation of the 5 h and 12 h groups from the control group (0 h) primarily along PC1, whereas the 20 h group largely overlapped with the control group, indicating greater similarity between these two groups in the multivariate expression space. PC2 contributed to the separation of samples according to the relative contribution of individual genes to this component, as illustrated by the loading vectors (Figure 1).

Table 3 delineates the transcriptional weight of each target gene within the first two principal components, encompassing their respective squared cosine (cos^2^) values and loading coefficients. Variations along PC1 were predominantly influenced by genes associated with epidermal barrier function and mucosal homeostasis, specifically Claudin12, DB-4, TGF-β1a, and MUC5AC. These markers demonstrated substantial contributions (ranging from 11.08% to 12.98%) and robust positive loadings (0.791–0.856), with elevated cos^2^ values (0.625–0.733) confirming their high representativeness within this axis. Conversely, PC2 was governed by the pro-inflammatory cytokine IL-1β, which uniquely accounted for 43.71% of the component variance, exhibiting a loading of 0.792 and a cos^2^ of 0.628. Secondary contributors to this second axis included TGF-β1b, Claudin8d, and Claudin7, with each providing approximately 13.8% of the input and displaying moderate loadings. Table 3 summarizes the contribution, loading coefficients, and cos^2^ values of each gene for the first two principal components. Claudin12, DB-4, TGF-β1a, and MUC5AC showed the largest contributions to PC1, whereas IL-1β contributed most strongly to PC2, followed by TGF-β1b, Claudin8d, and Claudin7. The corresponding loading coefficients and cos^2^ values indicate the relative contribution and representation of each gene within the two principal components. These results describe the statistical structure of the multivariate dataset and identify the genes contributing most strongly to each principal component.

### 3.4. Differential Gene Expression Analysis

Among the ten genes evaluated, eight genes showed significant differences in relative expression among treatments after Benjamini–Hochberg correction (adjusted *p* < 0.05). Significant treatment effects were detected for Claudin-7 (F = 5.48, adjusted *p* = 0.0072), Claudin-12 (F = 6.73, adjusted *p* = 0.0041), DB-4 (F = 3.90, adjusted *p* = 0.0304), IL-1β (F = 3.71, adjusted *p* = 0.0312), MUC2 (F = 3.04, adjusted *p* = 0.0489), MUC5AC (F = 7.84, adjusted *p* = 0.0029), TGF-β1a (F = 3.16, adjusted *p* = 0.0489), and TGF-β1b (F = 5.78, adjusted *p* = 0.0071). In contrast, IL-6 and Claudin-8d did not differ significantly among transport durations (Table 4).

**Figure 2:**
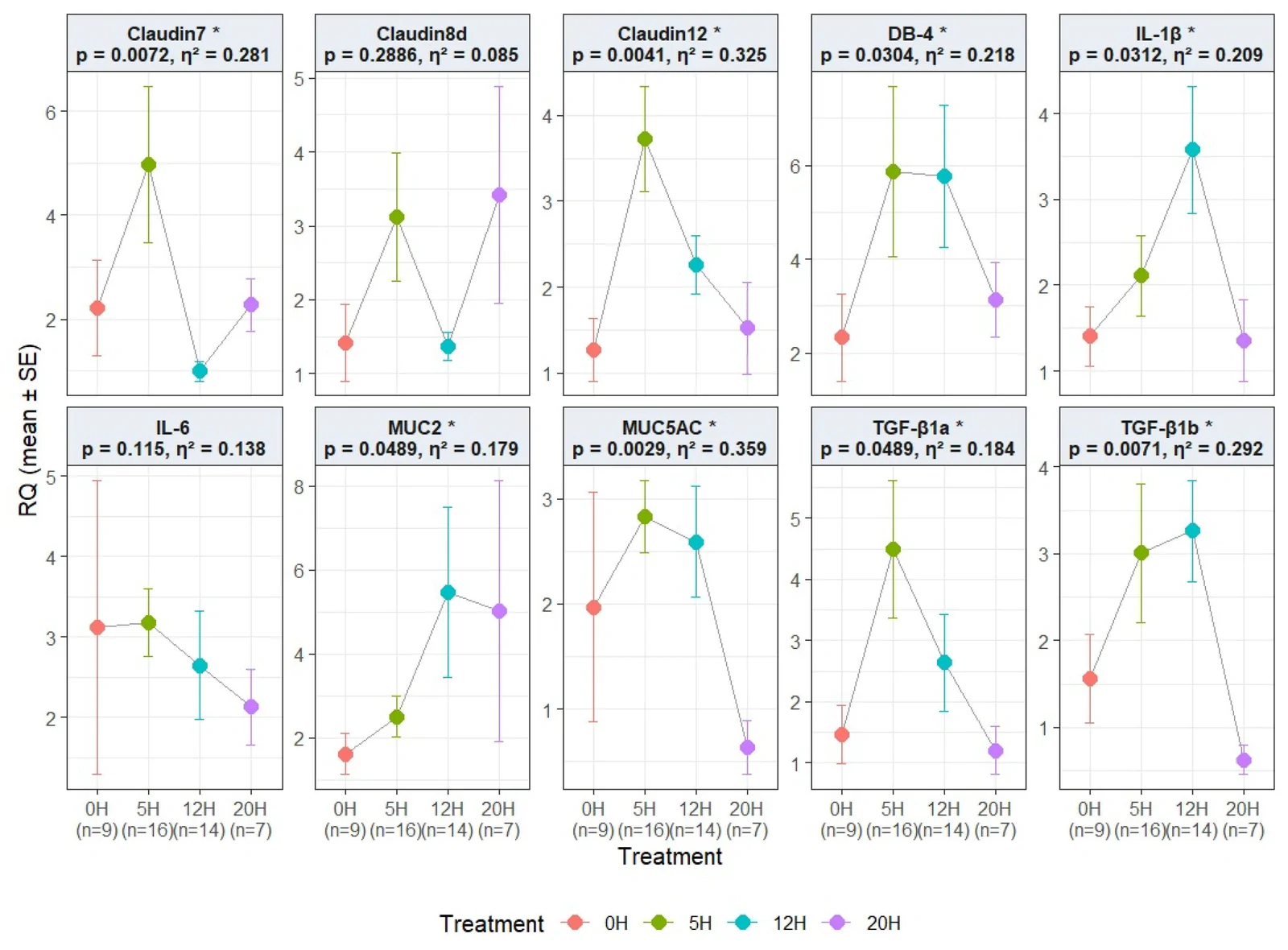
Mean ± SE of relative gene expression (RQ = 2^−ΔΔCt^) by treatment group. Asterisks denote genes with a significant treatment effect (BH-adjusted ANOVA). The IL6 value of sample 46 was excluded (NA).

Post hoc analyses revealed distinct temporal expression patterns among the evaluated genes. Claudin-7 and Claudin-12 showed early upregulation at 5 h, expression levels at later time points approached those observed in controls. Significant differences were observed between 0 h and 5 h for both genes, as well as between 5 h and subsequent sampling times. The inflammatory marker IL-1β exhibited maximal induction at 12 h, differing significantly from both the control and 20 h groups. Similarly, TGF-β1b expression increased during mid-stress exposure and declined by 20 h. Mucosal and epithelial barrier-associated genes also displayed pronounced temporal modulation. DB-4 and MUC2 showed significantly higher expression at 5 h and 12 h relative to controls, whereas MUC5AC expression decreased at 20 h compared with intermediate transport durations (Table 5 and Figure 2).

### 3.5. Co-Expression Analysis

**Figure 3:**
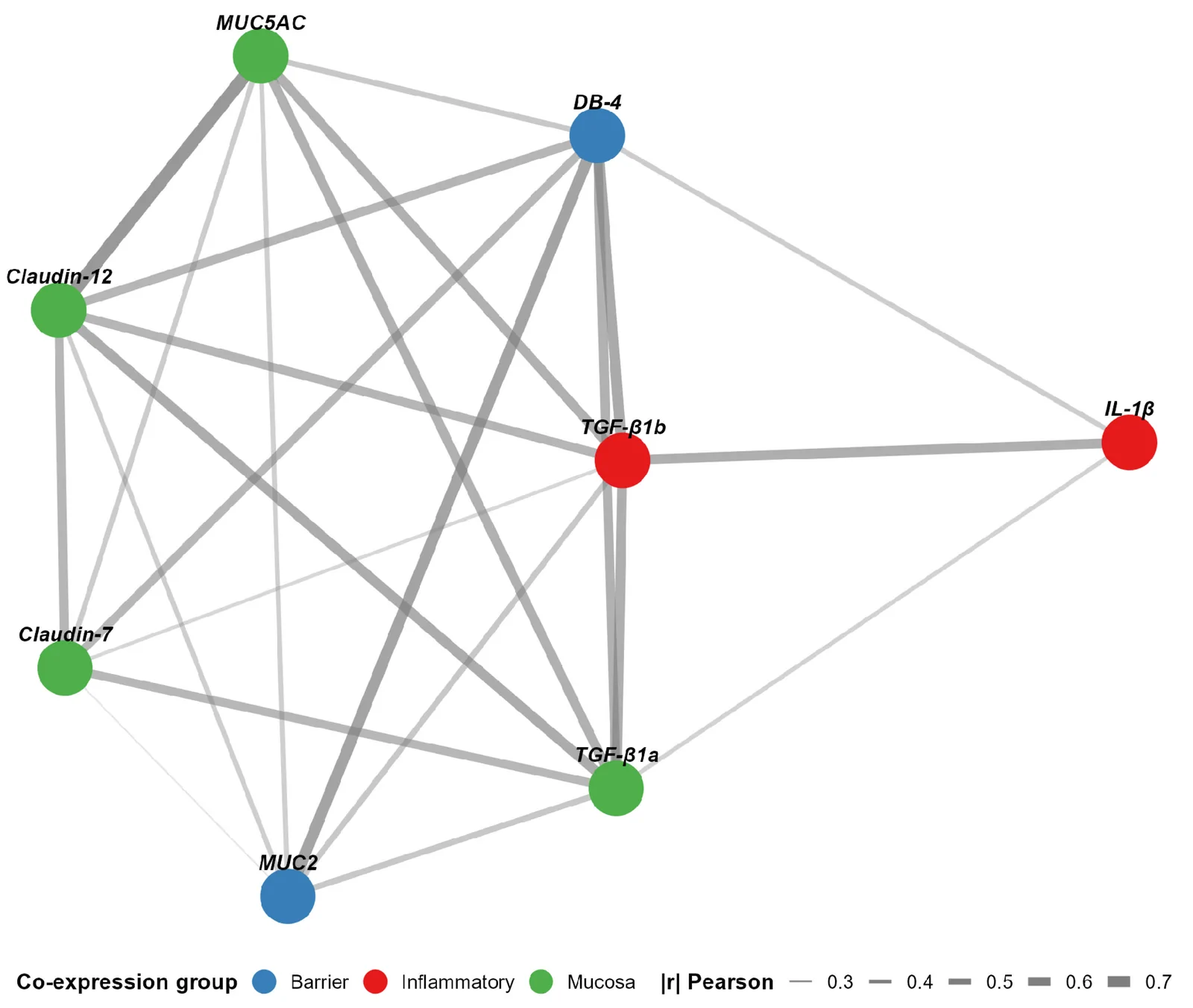
Correlation network of the eight significant genes. Nodes are colored according to co-expression modules (red: inflammatory; blue: barrier; green: mucosal). Edge width and opacity are proportional to the absolute value of the Pearson correlation coefficient. Only statistically significant correlations (*p* < 0.05) are displayed.

Hierarchical clustering analysis identified groups of correlated genes modules among the significantly regulated genes. The first module included the inflammatory genes IL-1β and TGF-β1b, which showed coordinated upregulation during the acute-phase response. The second module grouped DB-4 and MUC2, both associated with epithelial metabolism and mucus production. These genes exhibited similar quadratic expression kinetics, characterized by increased expression at 12 h followed by a reduction at 20 h. The third and largest module comprised Claudin-7, Claudin-12, MUC5AC and TGF-β1a, indicating coordinated regulation of tight-junction integrity and mucosal homeostasis. This module was characterized by early activation at 5 h and progressive normalization thereafter. The co-expression network further confirmed strong positive correlations among genes within each functional module, suggesting coordinated changes in expression patterns among these genes (Figure 3).

### 3.6. Polynomial Trend Analysis

Orthogonal polynomial contrasts were used to describe statistical temporal patterns in gene expression across the transport period. Significant quadratic components were detected for Claudin-12 (*p* = 0.0003), DB-4 (*p* = 0.0091), IL-1β (*p* = 0.0072), MUC5AC (*p* = 0.0001), TGF-β1a (*p* = 0.0149), and TGF-β1b (*p* = 0.0003), whereas significant linear components were identified for MUC2 (*p* = 0.0340) and MUC5AC (*p* = 0.0191). A significant cubic component was detected only for Claudin-7 (*p* = 0.0003). These polynomial components describe the statistical distribution of gene expressions across the sampling times (0, 5, 12, and 20 h) and should not be interpreted as definitive biological trajectories or underlying biological processes, particularly for the cubic component, which is more sensitive to individual variability (Table 6).

### 3.7. Effect of Treatments on Goblet Cells of the Epidermis

**Figure 4:**
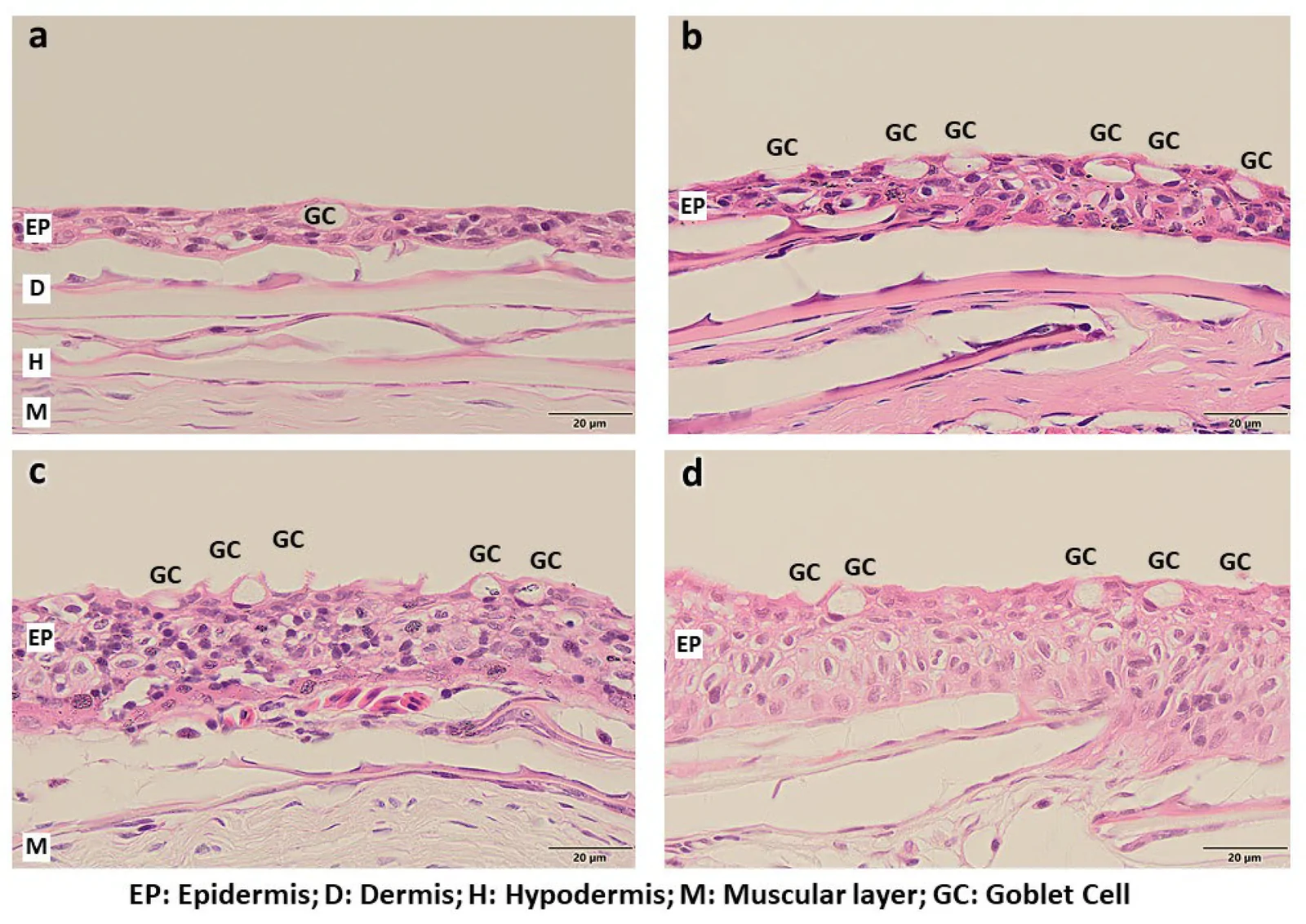
Histological sections of epidermal tissue from red tilapia (*Oreochromis* sp.) fry stained with hematoxylin–eosin at different experimental transport times. (**a**) Baseline samples at 0 h, (**b**) treatment at 5 h, (**c**) treatment at 12 h and (**d**) treatment at 20 h. Scale bar = 20 µm.

The epidermis contained few goblet cells (GC), which stained fuchsia-red with H&E, consistent with a basal state with minimal secretory hyperactivity. The overall organization of the tissue layers was preserved, although secretory activity appeared to be increased. Goblet cell density remained comparable during transport periods up to 12 h but decreased after 20 h, potentially reflecting an adaptive response to osmotic and mechanical stress associated with transport (Figure 4).

### 3.8. Descriptive Analysis of Goblet Cell Density

Goblet cell density was estimated to be 297 microscopic fields obtained from histological sections of individual fish. Multiple microscopic fields were examined to obtain a representative estimate of goblet cell density for each specimen; however, the mean goblet cell density calculated for each fish was used as the biological replicate in all statistical analyses. The microscopic fields were distributed as follows: 67 (0 h), 90 (5 h), 77 (12 h), and 63 (20 h).

Median goblet cell density values were 2.20 cells field^−1^ for the control group (0 h), 2.40 cells field^−1^ for the 5 h group, 2.27 cells field^−1^ for the 12 h group, and 1.50 cells field^−1^ for the 20 h group. Mean ± SD values followed a similar trend, decreasing from 2.55 ± 1.61 cells field^−1^ in the control group to 1.90 ± 1.41 cells field^−1^ in fish transported for 20 h (Table 7).

Goblet cell count and goblet cell density showed near-perfect collinearity (Spearman’s ρ = 0.976, *p* < 0.001), indicating that both variables captured the same underlying biological phenomenon. Consequently, goblet cell density was retained as the primary response variable for all subsequent analyses.

### 3.9. Effect of Transport Duration on Goblet Cell Density

Transport duration had a significant effect on the density of epidermal goblet cells (Kruskal–Wallis: H = 19.94; df = 3; *p* < 0.001). The associated effect size, expressed as epsilon squared, was

$$\varepsilon^{2}=0.06$$

indicating that transport duration explained a small-to-moderate effect size of the observed variation in goblet cell density.

**Figure 5:**
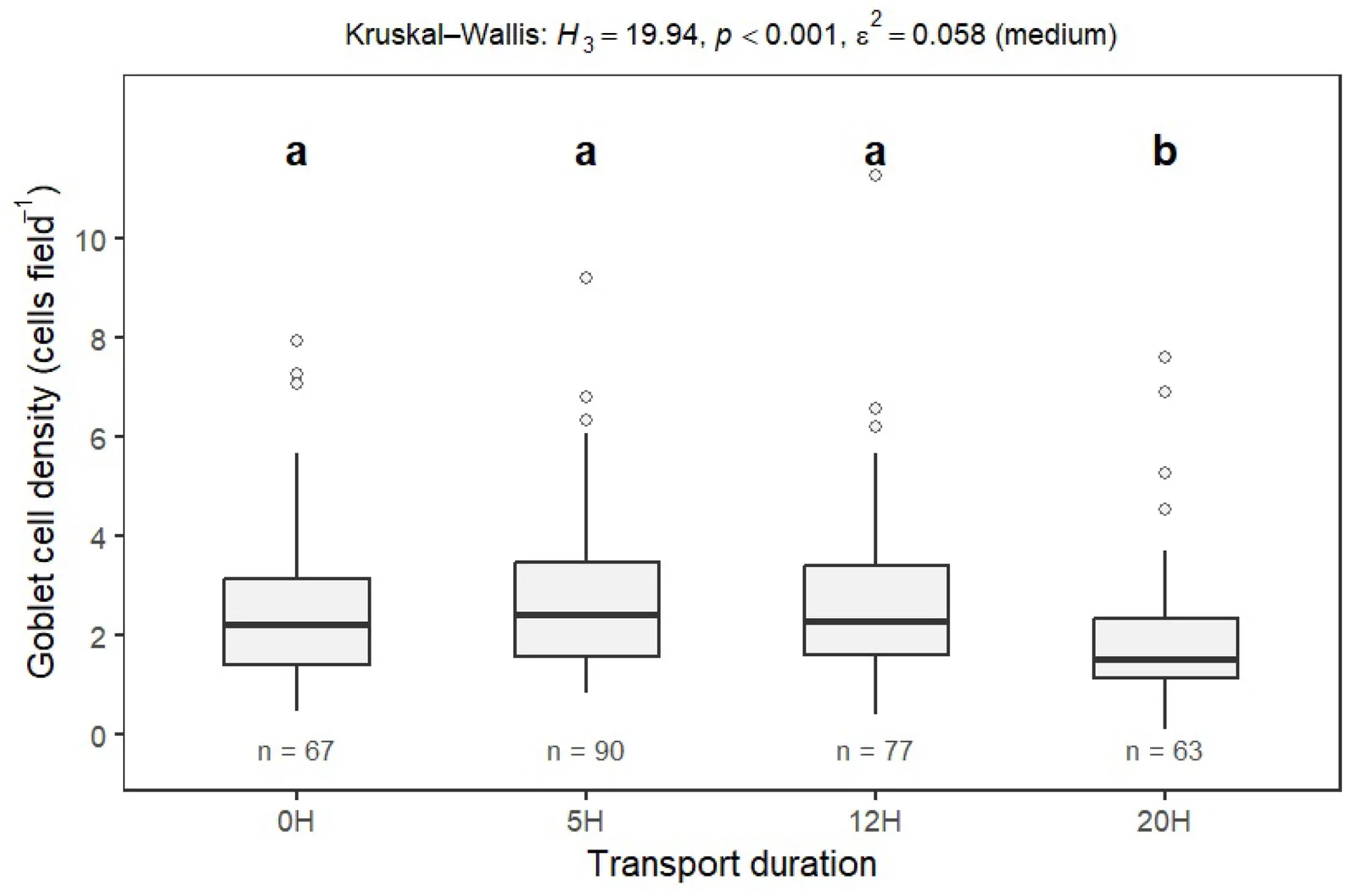
Goblet cell density (cells field^−1^) in the red tilapia (*Oreochromis* sp.) subjected to transport stress of varying durations. Boxes represent the interquartile range (Q1–Q3); the horizontal line indicates the median; whiskers extend to 1.5 × IQR; and open circles denote outliers. Different letters above the boxes indicate statistically significant differences between groups (Dunn–Bonferroni, *p* < 0.05). Sample sizes are shown below each box.

Post hoc pairwise comparisons using Dunn’s test with Bonferroni correction revealed that the 20 h group differed significantly from all other transport durations. Significant reductions in goblet cell density were detected between 20 h and 0 h (adjusted *p* = 0.0098), 20 h and 12 h (adjusted *p* = 0.0007), and 20 h and 5 h (adjusted *p* = 0.0001). In contrast, no significant differences were observed among the 0 h, 5 h, and 12 h groups (all adjusted *p* > 0.05). These results indicate that red tilapia maintained stable goblet cell density during short and moderate transport periods (≤12 h), whereas prolonged transport for 20 h induced a marked depletion of epidermal goblet cells (Table 8 and Figure 5).

The complete statistical outputs for goblet cell density are provided in Supplementary Tables S4 and S9.

### 3.10. Histomorphometric and Transcriptional Integration

The reduction in goblet cell density observed after 20 h of transport was consistent with the transcriptional patterns identified in the same experiment. Specifically, MUC2 and MUC5AC expression profiles exhibited non-linear trajectories, characterized by increased expression during intermediate stress periods followed by a decline at 20 h.

## 4. Discussion

### 4.1. Stress Gene Expression

Gene selection focused on genes associated with epidermal barrier integrity, mucus production, antimicrobial defense, and immune regulation, including mucins, tight-junction proteins, antimicrobial peptides, and cytokines. The aim was to understand how the epithelium maintains its barrier function during transport and how transport duration affects their expression.

PCA summarized the global expression patterns of the ten genes evaluated and allowed the identification of major biological axes associated with the transport response. The first two principal components explained 71.6% of total variance, indicating that these components captured a substantial proportion of the variation in the expression dataset, a value comparable to that reported for PCA of gene expression in *Danio rerio* exposed to different stressors, where the first two components explained on average 72–77% of the variation [22]. The PCA biplot showed partial separation of the 5 h and 12 h groups from the control (0 h) along PC1, whereas the 20 h group largely overlapped with the control, suggesting partial recovery of the transcriptional profile after prolonged stress exposure, likely associated with the activation of compensatory mucosal protection mechanisms that prevent sustained critical inflammation.

PC2 separated an inflammatory axis dominated by IL-1β and TGF-β1b from an epithelial barrier/mucus-production axis comprising claudins and MUC5AC, indicating that transport-induced responses in the epidermis are organized into distinct functional modules. This functional modularity is consistent with findings in *Oreochromis niloticus*, where transport in salt-free water increased epidermal IL-1β and TGF-β1 expression, whereas the addition of salt to the transport water attenuated these responses [23], and with studies in biofloc-reared *O. niloticus* in which IL-1β, TNF-α and LYZ were positively co-regulated in association with enhanced innate immunity [24]. Taken together, these results support PCA as a robust tool to visualize the global dynamics of the transport response, revealing both transient deviations and resilience.

Co-expression analysis using correlation matrices and hierarchical clustering, similar to the PCA approach, identified coordinated response modules among pro-inflammatory, mucus-producing, and epidermal barrier genes under transport stress in red tilapia fry. Therefore, the identification of co-expression patterns supports the presence of coordinated expression patterns among genes associated with inflammatory, mucus-related, and barrier functions, which can guide future studies including additional signaling and antimicrobial genes.

In the differential gene expression analysis, transport induced marked changes in tight junction genes (claudin 7, claudin 8d, and claudin 12), mucins (MUC2 and MUC5AC), antimicrobial peptides (DB-4), and cytokines (IL-1β, IL-6, TGF-β1a, and TGF-β1b). After 5 h of transport, Claudin-7 and Claudin-12 showed significantly higher expression than the control group, whereas their expression levels decreased at subsequent sampling times, approaching the values observed in the 0 h group. At 20 h, several transcripts returned toward baseline or declined, accompanied by reduced goblet cell density, suggesting that prolonged transport exceeded the adaptive capacity of the epidermal mucosa. These results are consistent with those reported by Zheng et al. [15], who showed that transport conditions activate TLR/NLR receptor pathways—key components of the innate immune system responsible for threat detection and defense activation in the epidermis—and also modulate mucus secretion, highlighting the role of epidermal tissue as a nonspecific barrier against handling stress. Similarly, in tilapia hybrids subjected to osmotic stress, a large number of genes related to ion transport and homeostasis were detected [25]. In this context, the epidermal response observed in red tilapia reinforces the concept of a rapid and targeted transcriptional reprogramming aimed at maintaining barrier integrity during transport.

Finally, the use of orthogonal polynomial contrasts made it possible to characterize temporal trajectories (linear, quadratic, and cubic) of the eight genes that showed significant differences during fry transport, taking advantage of the serial sampling at 0, 5, 12, and 20 h. Early peaks of MUC2 and the subsequent modulation of MUC5AC, claudins, and TGF-β indicate a transition from early mucus-associated transcriptional responses toward altered regulation of epithelial barrier-related genes. Similarly, time-series studies in Atlantic salmon subjected to acute stress have shown a progressive activation of immune genes with increasing time after stress exposure [26], whereas in C. elegans under heat stress a transcriptional tipping point has been described between 3–4 h that separates an initial dynamic phase from a more stagnant one [27]. In tilapia, these polynomial patterns support the view that the cutaneous response to transport is dynamic and phased, a key aspect for defining safe transport times and management strategies.

This study provides practical guidance for aquaculture by highlighting the central role of cutaneous mucus integrity during transport in maintaining fish welfare and reducing potential post-transport vulnerability. A deeper understanding of how transport affects the expression of immune-related genes will help inform strategies to mitigate stress-induced impairment of disease resistance in tilapia, thereby optimizing productivity and reducing economic losses.

### 4.2. Histological Evaluation of the Epidermis

Histological quantification of the epidermis was based on goblet cell (GC) density per microscopic field. The effect of the treatments on epidermal goblet cells revealed clear changes. In baseline sections (0 h of transport), the epidermis of red tilapia fry showed few GCs and therefore low secretory activity, whereas in the subsequent treatments (5 and 12 h) an increase in these cells in the epidermal tissue was observed, which may represent an adaptive morphological response associated with increased mucus-producing capacity.

In *O. niloticus* transported for 5 h in salt-free water, a similar pattern was observed, with epidermal thinning and a reduction in goblet cells, whereas fish transported in salted water showed significantly less reduction, and the tissue preserved goblet cell density and overall skin architecture [8,23]. Similarly, in fish subjected to capture and transport stress, a decrease in goblet cells in the skin and gills has been reported, clearly potentially increasing susceptibility to opportunistic infections [24]. Together, these findings reinforce that goblet cells are highly sensitive both to transport stress and to changes in water conditions and support the interpretation of GC density as a viable histomorphological indicator to evaluate the functional capacity of the epidermal barrier.

The results also revealed that, after the increases observed at 5 and 12 h of transport as a defensive mechanism, there was a significant decrease at 20 h, reflecting deterioration of the protective capacity of the epidermis. This pattern is consistent with the findings of Szakolczai [24] and Sadek-Hana et al. [8], who reported hyperplasia–depletion dynamics in the epidermis and gills of fish subjected to capture and transport stress. The decrease in GC density after 20 h of transport was in line with the transcriptional dynamics of mucins and barrier genes. MUC2 and MUC5AC showed non-linear trajectories, with increased expression during intermediate stress periods and a decline at 20 h, suggesting an initial phase of mucus hypersecretion followed by reduction in mucus-associated transcriptional activity and goblet cell density.

This histomorphometric–transcriptional integration indicates that transport durations ≤ 12 h appear to remain within the adaptive capacity of the cutaneous mucosa, whereas 20 h was associated with reduced indicators of epidermal mucosal defense. A similar coupling between GC morphology and MUC2/MUC5AC expression was also reported by Sadek-Hana et al. [8] during 5 h transport, with higher mucin expression and limited GC opening in salt-free water and preservation of surface integrity together with increased GC openings when NaCl was added.

## 5. Conclusions

Transport conditions significantly modulated the expression of tight junction, mucin, antimicrobial peptide and cytokine genes, with early peaks of MUC2 and subsequent activation of MUC5AC, claudins and TGF-β indicating a transition from an early mucus-associated transcriptional response toward altered regulation of epithelial barrier-related genes, characterized toward increased expression of genes associated with epithelial barrier regulation.

Histomorphometric analysis of the epidermis demonstrated temporal changes in goblet cell density, with higher values during the early transport periods and a significant reduction after 20 h of transport. A similar temporal pattern was observed for mucus-associated gene expression, which also declined at 20 h. Taken together, integration of transcriptional data and goblet cell dynamics suggest that transport durations shorter than 12 h remain within the adaptive capacity of the mucosal response, whereas 20 h of transport is associated with reduced indicators of epidermal mucosal defense.

## Figures and Tables

**Table 1:** Primers used in this study for qPCR.

Gene	Primer *	Sequence (5′–3′)
Claudin7	Claudin7F	CGTCCTGCTGATTGGATCTC
	Claudin7R	CAAACGTACTCCTTGCTGCTG
Claudin8d	Claudin8dF	GCAGTGTAAAGTGTACGACTCTCTG
	Claudin8dR	CACGAGGAACAGGCATCC
Claudin12	Claudin12dF	CTTCATCATCGCCTTCATCTC
	Claudin12dR	GAGCCAAACAGTAGCCCAGTAG
MUC2	OmMUC2F	CCAGGCACAGAAAAGACAGATGC
	OmMUC2R	GGATGTAGGAGTGCTTGACC
MUC5AC	OmMUC5ACF	GCTCTGGTCTTCGGACTATCTG
	OmMUC5ACR	GCTGCTCTTACACAACGACG
DB-4	omDB4F	GCAACTCTTCTAAAGAACAGT
	omDB4R	CGTGGGCGACACAGCATACAAATCC
IL-1β	IL1βF	ACATTGCCAACCTCATCATCG
	IL1βR	TTGAGCAGGTCCTTGTCCTTG
IL-6	IL6F	ACTCCCCTCTGTCACACACC
	IL6R	GGCAGACAGGTCCTCCACTA
TGF-β1a	TGFβ1aF	CTCACATTTTACTGATGTCACTTCCTGT
	TGFβ1aR	GGACAACTGCTCCACCTTGTG
TGF-β1b	TGFβ1bF	CATGTCCATCCCCCAGAACT
	TGFβ1bR	GGACAACTGTTCCACCTTGTGTT
β-actin	Actin F Actin R	ATTCTCTGGTGGGCTCACTG AGCCCTATGCACAAACCTTCA

* F indicates the forward orientation and R the reverse orientation.

**Table 2:** Water quality parameters measured using a multi-parameter probe at the beginning and end of the transportation periods for tilapia fry (*Oreochromis* sp.).

Parameter	5 H		12 H		20 H	
	Initial	Final	Initial	Final	Initial	Final
Temperature (°C)	31.05 ± 0.13 a	26.80 ± 1.42 c	27.89 ± 0.09 c	26.77 ± 0.62 c	29.13 ± 0.98 bc	28.92 ± 0,17 b
pH	6.49 ± 0.25 ab	5.94 ± 0,58 b	6.74 ± 0.08 a	6.28 ± 0.07 ab	6.38 ± 0.15 ab	6.16 ± 0.24 b
DO (mg/L)	6.10 ± 0. 12 e	13.76 ± 0.36 a	7.42 ± 0.34 d	11.98 ± 1,41 b	7.36 ± 0.26 d	9.40 ± 0.61 c
Oxygen Saturation (%)	95.30 ± 3.80 e	180.95 ± 2.18 a	97.05 ± 0.95 d	151.45 ± 4,29 b	97.12 ± 0.95 d	122.62 ± 6.36 c
Electrical conductivity (µS/cm)	44.00 ± 2.65 d	105.98 ± 6.31 c	30.13 ± 0.92 e	249.46 ± 12.84 b	32.73 ± 0.49 f	378.17 ± 0.87 a
TDS (mg/L)	22.00 ± 2.29 d	52.55 ± 2.64 c	15.27 ± 2.94 de	125.60 ± 9.74 b	7.03 ± 0.52 e	190.39 ± 7.92 a
Alkalinity (mg/L)	40.00 ± 5.57 e	52.48 ± 1.91 d	75.03 ± 0.77 c	163.76 ± 3.61 b	29.69 ± 0.87 f	197.89 ± 0.15 a

Data corresponds to the meaning of the replicates ± SD. Different letters (a–f) suggest statistically significant differences.

**Table 3:** Contribution of each gene to the two principal components estimated to measure their relationship with transport stress in red tilapia fry.

Gen	Contribution PC1	Contribution PC2	Cos2 PC1	Cos2 PC2	Loading PC1	Loading PC2
Claudin12	12.98	1.62	0.733	0.023	0.856	−0.152
DB-4	12.48	1.98	0.705	0.028	0.840	0.169
TGF-β1a	11.99	0.90	0.677	0.013	0.823	0.114
MUC5AC	11.08	0.99	0.625	0.014	0.791	−0.119
TGF-β1b	10.94	13.81	0.618	0.198	0.786	0.445
IL-6	10.39	6.07	0.587	0.087	0.766	−0.295
Claudin7	9.06	13.75	0.511	0.197	0.715	−0.444
MUC2	8.93	3.39	0.504	0.049	0.710	0.221
Claudin8d	8.86	13.79	0.500	0.198	0.707	−0.445
IL-1β	3.29	43.71	0.186	0.628	0.431	0.792

**Table 4:** One-way ANOVA results for each gene. F-statistic, raw *p*-value, and Benjamini–Hochberg adjusted *p*-value are reported. Genes marked ‘ns’ were not significant after correction.

Gene	F	*p*-Value	*p*-adj (BH)	Significance
Claudin7	5.48	0.0029	0.0072	**
Claudin8d	1.30	0.2886	0.2886	ns
Claudin12	6.73	0.0008	0.0041	**
DB-4	3.90	0.0152	0.0304	*
IL-1 β	3.71	0.0187	0.0312	*
IL-6	2.19	0.1035	0.1150	ns
MUC2	3.04	0.0391	0.0489	*
MUC5AC	7.84	0.0003	0.0029	**
TGF-β1a	3.16	0.0346	0.0489	*
TGF-β1b	5.78	0.0021	0.0071	**

* *p* < 0.05; ** *p* < 0.01; ns = not significant.

**Table 5:** Significant Tukey post hoc contrasts (*p* < 0.05) between treatment groups for the eight significant genes. Estimates are on the log_2_(RQ) scale.

Gene	Contrast	Estimate	SE	*p*-Value
Claudin7	0H–5H	−1.641	0.5843	0.0364
Claudin7	5H–12H	1.934	0.5132	0.0027
Claudin12	0H–5H	−1.649	0.4455	0.0033
Claudin12	5H–20H	1.749	0.4845	0.0043
DB-4	0H–5H	−1.852	0.6253	0.0248
DB-4	0H–12H	−2.032	0.6411	0.0146
IL-1 β	0H–12H	−1.466	0.5469	0.0493
IL-1 β	12H–20H	1.668	0.5925	0.0358
MUC2	0H–12H	−1.719	0.5766	0.0237
MUC5AC	5H–20H	2.874	0.6164	0.0002
MUC5AC	12H–20H	2.311	0.6297	0.0036
TGF-β1b	5H–20H	2.028	0.6153	0.0104
TGF-β1b	12H–20H	2.394	0.6285	0.0024

SE = Standard error.

**Table 6:** Polynomial contrast results for the eight significant genes. Estimates are on the log_2_(RQ) scale.

Gene	Contrast	Estimate	SE	T-Ratio	*p*-Value	Sig
Claudin7	linear	0.7289	2.1812	0.3342	0.7399	ns
Claudin7	quadratic	−0.4601	0.8733	−0.5269	0.6011	ns
Claudin7	cubic	6.6894	1.6939	3.9491	0.0003	***
Claudin12	linear	−1.0346	1.6630	−0.6221	0.5372	ns
Claudin12	quadratic	−2.6624	0.6658	−3.9985	0.0003	***
Claudin12	cubic	2.1054	1.2915	1.6302	0.1105	ns
DB-4	linear	4.1674	2.3342	1.7853	0.0814	ns
DB-4	quadratic	−2.5544	0.9346	−2.7331	0.0091	**
DB-4	cubic	0.7915	1.8128	0.4366	0.6646	ns
IL-1 β	linear	0.2771	1.9911	0.1392	0.8900	ns
IL-1 β	quadratic	−2.2529	0.7972	−2.8259	0.0072	**
IL-1 β	cubic	−2.8457	1.5463	−1.8403	0.0728	ns
MUC2	linear	4.6002	2.0993	2.1913	0.0340	*
MUC2	quadratic	−1.4152	0.8406	−1.6836	0.0997	ns
MUC2	cubic	−0.9086	1.6304	−0.5573	0.5803	ns
MUC5AC	linear	−5.1579	2.1158	−2.4377	0.0191	*
MUC5AC	quadratic	−3.6531	0.8472	−4.3122	0.0001	***
MUC5AC	cubic	0.1545	1.6432	0.0940	0.9255	ns
TGF-β1a	linear	−1.7794	2.3675	−0.7516	0.4565	ns
TGF-β1a	quadratic	−2.4073	0.9479	−2.5396	0.0149	*
TGF-β1a	cubic	2.4366	1.8386	1.3253	0.1922	ns
TGF-β1b	linear	−2.8955	2.1121	−1.3709	0.1777	ns
TGF-β1b	quadratic	−3.3348	0.8457	−3.9435	0.0003	***
TGF- β1b	cubic	−2.1866	1.6403	−1.3331	0.1897	ns

SE = Standard error, Sig = Significance level. * *p* < 0.05; ** *p* < 0.01; *** *p* < 0.001; ns = not significant.

**Table 7:** Descriptive statistics of goblet cell density (cells field^−1^) in red tilapia by transport duration. Values are presented as Median (Q1–Q3) and Mean ± SD.

Transport Duration	*n*	Median (Q1–Q3)	Mean ± SD	Min	Max
0 H	67	2.2 (1.38–3.13)	2.55 ± 1.61	0.46	7.93
5 H	90	2.4 (1.57–3.45)	2.72 ± 1.52	0.83	9.20
12 H	77	2.27 (1.58–3.4)	2.67 ± 1.67	0.38	11.27
20 H	63	1.5 (1.11–2.33)	1.9 ± 1.41	0.07	7.60

*n* = Sample size, (Q1–Q3) = Interquartile range.

**Table 8:** Dunn’s pairwise post hoc test with Bonferroni correction. Significant differences (*p* < 0.05) are highlighted.

Comparison	Z Statistic	*p*-adj (Bonf.)	Significant
0 H–12 H	−0.653	1.0000	No
0 H–20 H	2.943	0.0098	Yes
12 H–20 H	3.682	0.0007	Yes
0 H–5 H	−1.033	0.9054	No
12 H–5 H	−0.370	1.0000	No
20 H–5 H	−4.158	0.0001	Yes

## Data Availability

The original contributions presented in this study are included in the article/Supplementary Materials. Further inquiries can be directed to the corresponding author.

## Supplementary Materials

The following supporting information can be downloaded at: https://www.mdpi.com/article/10.3390/ani16162539/s1, Table S1: goblet_cell_report.qmd; Table S2: tilapia_stress_gene_expression.qmd; Table S3: analisis_estres_tilapia. R; Table S4: Statistical_report_goblet_density.docx; Table S5: base HyE globets.xlsx; Table S6: Datos Animals.xlsx; Table S7: Datos Genes Tilapia.xlsx; Table S8: Datos histopatologia.xlsx; Table S9: Statistical_results_goblet_density.xlsx.

## Abbreviations

The following abbreviations are used in this manuscript:

MUC	Mucin
TGF-β1a and TGF-β1b	Anti-inflammatory cytokines
IL	Interleukin
GC	Goblet cells
H&E	hematoxylin and eosin
