# Supplementary material for: Temporal Influence of Transport Length on Epidermal Barrier Integrity, Mucin Transcription, and Goblet Cell Kinetics in Red Tilapia (Oreochromis sp.) Fry
Source: Animals (Basel). 2026 Aug 14;16(16):2539. doi: 10.3390/ani16162539 (PMC13508840; doi:10.3390/ani16162539)
Supplement: Supplementary file 1 [file animals-16-02539-s001.zip › Table S4. Statistical_report_goblet_density.pdf]

1. STATISTICAL REPORT — GOBLET CELL DENSITY IN RED TILAPIA UNDER TRANSPORT STRESS

1.1. METHODS SECTION

2.X. Statistical analysis Goblet cell density (cells field^{-1}) was used as the primary response variable. Prior to inferential testing, the normality of the distribution within each treatment group was assessed using the Shapiro–Wilk test, and homogeneity of variances was evaluated with Levene's test. Because normality was violated in all groups (all $W < 0.884$, $p < 0.001$), a non-parametric Kruskal–Wallis one-way analysis of variance by ranks was performed to assess the effect of travel duration (0, 5, 12, and 20 h) on goblet cell density. Effect size was estimated using epsilon squared (ε^2), with reference thresholds of small ($\varepsilon^2 \geq 0.01$), medium ($\varepsilon^2 \geq 0.06$), and large ($\varepsilon^2 \geq 0.14$) (Tomczak & Tomczak, 2014). Pairwise comparisons were conducted using Dunn's test with Bonferroni correction for multiple comparisons. Statistical significance was set at $\alpha = 0.05$. All analyses were performed in R (v4.5.2) using the packages car (Fox & Weisberg, 2019) and dunn.test (Dinno, 2017).

1.2. RESULTS SECTION

3.X. Goblet cell density Levene's test confirmed homogeneity of variances among groups ($F_{3,293} = 0.832$, $p = 0.477$). However, the Shapiro–Wilk test indicated significant departures from normality within each group (0H: $W = 0.883$, $p < 0.001$; 5H: $W = 0.891$, $p < 0.001$; 12H: $W = 0.840$, $p < 0.001$; 20H: $W = 0.795$, $p < 0.001$); therefore, a Kruskal–Wallis test was applied. Goblet cell density differed significantly among travel duration groups ($H_3 = 19.94$, $p < 0.001$, $\varepsilon^2 = 0.058$, medium effect). Post-hoc Dunn–Bonferroni comparisons revealed that the 20-h group showed significantly lower goblet cell density than the 0-h ($p = 0.010$), 5-h ($p < 0.001$), and 12-h ($p < 0.001$) groups. No significant differences were detected among the 0-h, 5-h, and 12-h groups (all $p \geq 0.905$). Median (Q1–Q3) goblet cell densities were: 0H = 2.20 (1.47–3.00), 5H = 2.40 (1.67–3.40), 12H = 2.27 (1.47–3.27), and 20H = 1.50 (0.77–2.27) cells field^{-1} (Figure X).

1.2.1. Table X. Descriptive statistics

Table X. Descriptive statistics of goblet cell density (cells field^{-1}) in red tilapia intestine by travel duration.

Travel duration	n	Median (Q1–Q3)	Mean ± SD
0H	67	2.2 (1.38–3.13)	2.55 ± 1.61
5H	90	2.4 (1.57–3.45)	2.72 ± 1.52
12H	77	2.27 (1.58–3.4)	2.67 ± 1.67
20H	63	1.5 (1.11–2.33)	1.9 ± 1.41

Note: Different letters indicate statistically significant differences between groups (Dunn–Bonferroni, $p < 0.05$). Levene: $F_{3,293} = 0.832$, $p = 0.477$. Kruskal–Wallis: $H_3 = 19.94$, $p < 0.001$, $\varepsilon^2 = 0.058$.
